# Therapeutic approaches and drug-resistance in chronic lymphocytic leukaemia

**DOI:** 10.20517/cdr.2019.111

**Published:** 2020-05-11

**Authors:** Narjis Fatima, Kyle R. Crassini, Lauren Thurgood, Yandong Shen, Richard I. Christopherson, Bryone Kuss, Stephen P. Mulligan, Oliver Giles Best

**Affiliations:** ^1^Kolling Institute of Medical Research, University of Sydney, Royal North Shore Hospital, Sydney, NSW 2065, Australia.; ^2^School of Life and Environmental Sciences, University of Sydney, Sydney NSW 2008, Australia.; ^3^College of Medicine and Public Health, Flinders University, Bedford Park, Adelaide, SA 5042, Australia.

**Keywords:** Chronic lymphocytic leukaemia, drug resistance, targeted cancer therapy

## Abstract

The treatment of chronic lymphocytic leukaemia has been revolutionised in recent years, first by the introduction of chemoimmunotherapy regimens and subsequently by the development of drugs, including ibrutinib, idelalisib and venetoclax, that target components of the B-cell receptor signalling pathway or B-cell lymphoma 2 family of proteins. Despite high initial response rates in patients treated with chemoimmunotherapy or targeted agents, a significant proportion of patients relapse with progressive and refractory disease. In a subset of these patients, drug resistance has been associated with specific genetic lesions or activation of alternate pro-survival pathways. However, the mechanisms that confer drug resistance in the remainder of the patients with refractory disease have yet to be fully elucidated. In this review, we discuss our current understanding of the mechanics of drug resistance in chronic lymphocytic leukaemia and describe how this knowledge may aid in rationalising future treatment strategies to prevent the development of refractory or aggressive transformation of the disease.

## Introduction

Chronic lymphocytic leukaemia (CLL) is the most common leukaemia among adults in the Western world. The disease is characterised by the accumulation of apoptosis-resistant B lymphocytes in the blood, bone marrow, lymph nodes and spleen. The bone marrow and lymph nodes play a significant role in the pathogenesis and drug sensitivity of CLL. Within these tissues the leukaemic cells interact with a variety of cells, including stromal cells, T cells and adipocytes, forming pseudofollicular structures known as proliferation centres. CLL cells within proliferation centres are phenotypically distinct from circulating CLL cells with antigen expression changes consistent with B cell activation and proliferation^[1]^.

Despite significant advances in the treatment of CLL in recent years, the disease is still widely considered incurable. In many patients, drug resistance, relapse and disease progression or aggressive transformation have been associated with specific genetic lesions. The most well described of these lesions is deletion of the region on the short arm of chromosome 17 (del17p), which encompasses the *TP53* gene. Under normal cellular conditions, expression of the TP53 protein is low but is induced by DNA damage, resulting in a signalling cascade that triggers cell cycle arrest and DNA repair or apoptosis. The importance of TP53 in cancer is highlighted by the fact that over 50% of all cancers harbour mutations in the *TP53* gene. In CLL, as in other cancers, *TP53* lesions are strongly associated with poor outcome following many of the current therapeutic strategies.

Deletions or mutations of the ataxia telengiectasia mutated (*ATM*) gene are also common among CLL patients and are associated with intermediate risk disease^[2,3]^. ATM is upstream of TP53 in signalling the response of cells to DNA damage, acting as a “sensor” of DNA breaks. Following DNA damage, ATM is activated and phosphorylates the ubiquitin ligase MDM-2, which dissociates from TP53. This stabilises the TP53 protein enabling it to translocate from the cytoplasm into the nucleus where it functions as a transcription factor regulating the expression of multiple pro-apoptotic or cell cycle regulators. Deletions of *TP53* or *ATM*, in the 17p13 and 11q22-q23 chromosomal regions, respectively, are identified in approximately 4%-10% (*TP53*) and 10%-20% (*ATM*) of patients at diagnosis, but the proportion increases to around 40% (*TP53*) in patients with relapsed/refractory disease^[4]^. Earlier studies suggested that fludarabine-based chemoimmunotherapy (CIT) regimens may overcome the poor prognosis of patients with *ATM* deletions^[5]^; however, the German CLL8 trial^[6]^ and a recent study suggests that these patients have significantly shorter progression-free survival duration^[7]^.

A better understanding of the mechanisms that drive CLL-cell survival and proliferation, particularly within the tumour microenvironment, has led to the development of novel, more targeted therapies. However, as data from trials of these newer agents accumulate, it has become increasingly evident that a significant proportion of patients still relapse with progressive disease. High throughput genetic sequencing techniques, such as next-generation sequencing, have led to a rapid increase in our understanding of the genetic diversity of CLL. The ability to monitor patients from diagnosis through treatment to relapse have shown the marked clonal evolution that occurs in CLL^[8]^. As highlighted, *TP53* lesions are shown to occur more frequently in a relapsed and refractory setting and remain strong predictors of poor outcome. However, more sensitive sequencing techniques have shown that exposure to novel therapeutic agents can result in the emergence of clones harbouring additional molecular lesions, many of which may be associated with drug resistance. Identifying effective therapeutic approaches, particularly for poor-risk disease patients, remains an important area of research for CLL. Elucidating the mechanisms of drug resistance in CLL is key in the development of novel therapeutic approaches that will improve patient outcomes and mitigate the risk of drug resistance.

This review discusses the significant advances in CLL therapy that have occurred in the last two decades and describes our current understanding of the mechanisms of drug resistance in this disease.

## CIT

CIT refers to therapy regimens incorporating both chemotherapeutic drugs and antibodies that bind to specific antigens on cancer cells. The development of anti-CD20 antibodies and incorporation of these into chemotherapy regimens represented a significant milestone in the treatment of CLL^[9,10]^. CIT regimens are associated with significantly higher response rates compared to chemotherapy-only regimens^[10-12]^ and CIT remains a commonly used front-line treatment option for CLL.

Fludarabine and cyclophosphamide in combination with the anti-CD20 monoclonal antibody rituximab (FCR) remains the most widely utilised of the CIT regimens in CLL; initial trials demonstrated that the addition of rituximab to the FC regimen was associated with a significant improvement in both progression-free survival and overall survival. FCR has proven to be an effective therapy for many patients, particularly those with mutated *IGHV*, with half of these patients remaining in permanent complete remission^[13]^. However, FCR therapy is commonly associated with neutropenia, an increased risk of infection and an increased risk of treatment-related myelodysplasia and acute myeloid leukaemia^[14]^.

The mechanisms of action of rituximab have been reported to include antibody-dependent cellular cytotoxicity (ADCC), complement-dependent cellular cytotoxicity (CDC), chemo-sensitisation, direct cytotoxicity and antibody dependent cellular phagocytosis, although the contribution of these effects to the overall efficacy of rituximab appear to be disease dependent. There are direct effects of rituximab on the translocation of the CD20 molecule in the plasma membrane-associated lipid rafts, which results in increased calcium flux and caspase-dependent induction of apoptosis^[15]^. We and others have confirmed that this redistribution occurs in CLL cells following treatment with rituximab^[16,17]^.

Resistance to CIT in CLL arises due to the presence or acquisition of a specific lesion(s) that is present in a subclone of CLL cells rendering these cells insensitive to therapy. During or after therapy, evolution or selection of these resistant clones occurs. Such lesions commonly arise in the DNA damage-response pathway, in the *ATM* and *TP53* genes. Patients with *TP53* lesions of in particular generally respond poorly to FCR or relapse early with aggressive disease following treatment^[3,6,18]^. This occurs largely because the efficacy of the FCR regimen is dependent on the ability of the leukaemic cells to mount a DNA-damage response, which under normal circumstances triggers apoptosis or cell cycle arrest. In patients with lesions in *ATM* or *TP53*, DNA damage induced by these drugs does not trigger an apoptotic signalling cascade or block cell proliferation. While the prognostic significance of high-frequency deletions or mutations of *TP53* is well established, the impact of low frequency lesions remains unclear. In a recent study of newly diagnosed CLL patients, Brieghel *et al*.^[19]^ found that the presence of *TP53* mutations with a variant allele frequency of > 10% in the absence of *TP53* deletion had no impact on overall survival or treatment-free survival. However, in patients requiring treatment, the presence of *TP53* mutations with variant allele frequencies of > 1% were of prognostic significance. As the authors suggested, these data indicated the need for further studies to define the level at which *TP53* mutations are clinically significant.

Several studies have identified associations between resistance to fludarabine and rituximab and microRNA (miR) expression in CLL cells. Resistance to fludarabine has been linked to increased expression of miRs-148a, 222 and 21^[20]^ and rituximab resistance has been associated with decreased expression of miRs-125b and 532-3p^[21]^. While many miRs are regulated by *TP53*, Cerna *et al*.^[22]^ demonstrated that expression of miR-34a has independent prognostic significance, suggesting that low expression of miR-34a may confer resistance to FCR in a subset of patients with no lesion of either *ATM* or *TP53*. The same study also showed that increased miR-34a expression following FCR-induced DNA damage represses the transcriptional activity of FOXP1 and results in a reduction of B cell receptor (BCR)-mediated signalling. This suggests that while CIT regimens do not directly inhibit intracellular signalling, the efficacy of FCR in CLL may depend on indirect inhibition of kinases in the BCR pathway.

Next-generation sequencing techniques have enabled detailed examination of the genome of CLL cells and have highlighted a number of high frequency genetic variants including *NOTCH1*, *SF3B1* and *BIRC3*. Mutations of *NOTCH1* have been associated with decreased levels of CD20 via a mechanism involving histone deacetylase-mediated repression of CD20 expression, resulting in reduced sensitivity to CD20-targeted therapies *in vitro*^[23]^. Furthermore, patients with mutations of *NOTCH1* received no benefit from addition of rituximab to FC in the CLL8 trial and a weak benefit from addition of ofatumumab to chlorambucil in the phase III trial COMPLEMENT 1^[24]^, suggesting that mutations of *NOTCH1* may confer reduced sensitivity or resistance to therapy with anti-CD20 monoclonal antibodies.

Longitudinal studies on CLL patients who relapse following FCR treatment suggest that clonal selection and evolution both occur. Clonal selection occurs when lesions are evident, often as minor clones, prior to treatment but predominate following treatment. Clonal evolution differs in that lesions are not present prior to therapy but are induced in cells by the genotoxic nature of the treatment. Genetic damage induced by treatment may subsequently confer a survival advantage and trigger the propagation of a new, more aggressive, disease clone. This was demonstrated in an analysis of patients enrolled in the German CLL8 trial, in which copy number changes were twice as likely following treatment with FC or FCR than over a similar period prior to treatment^[25]^. Studies have also demonstrated that accumulation of genetic aberrations and increased genetic complexity resulting from chromosomal instability are associated with poor outcome and response to treatment^[26,27]^.

Trials of other CIT regimens, including bendamustine in combination with rituximab (BR) demonstrate this is also an effective and well-tolerated treatment regimen, even among elderly patients, with an overall response rate of 45.5%^[28]^. Treatment with BR is associated with a significantly lower incidence of neutropenia than FCR (19.7% in BR-treated compared to 34% in FCR-treated patients). However, unmutated *IGHV* gene status and/or deletions of chromosome 17p remained strong indicators of poor response or outcome following treatment with BR. Furthermore, unlike FCR, there is no plateau in the survival curve of *IGHV*-mutated patients treated with BR. Similar to fludarabine, patients with deletions or mutations of *TP53*, have poor outcomes following treatment with bendamustine due to the dependence of the drug on the TP53-mediated apoptotic cascade^[28]^. Factors associated with resistance to the FCR and BR regimens are detailed in Table 1.

**Table 1:** Details of the features associated with resistance or poor response of CLL patients to current therapeutic drugs or regimens

Drug/Regimen	Drug target(s)	Features independently associated with resistance or poor response to treatment	Ref.
FCR	Genotoxic and anti-CD20 monoclonal antibody	Deletion of 17p13 (*TP53*)	[3,18]
		Deletion of 11q22-23 (*ATM*)	[3,7]
		*TP53* or *ATM* mutations	[6,7]
		*NOTCH1, BIRC3, SF3B1, RPS15* mutations	[6,119]
		Increased expression of miRs 148a, 222 and 21 (fludarabine)	[20]
		Decreased expression of miRs 125b and 532-3p (rituximab)	[21]
		Low miR34a expression	[22]
		Unmutated *IgHV* status	[13,18,120]
BR	Genotoxic and anti-CD20 monoclonal antibody	Deletion of 17p13 (*TP53*)	[121]
		Deletion of 11q22-23 (*ATM*)	[7]
		Unmutated *IgHV* status	[122]
Ibrutinib	BTK	*BTK* mutations (C481 or V5371)	[46,47]
		*PLC-g2* mutations	[46]
		PD-1 overexpression	[50]
		Pro-survival shift in BCL-2 protein expression	[50,52]
		Increased AKT/mTOR signalling	[50]
		Deletion of 8p and decreased TRAIL-R expression	[123]
Acalabrutinib	BTK	*BTK* mutations (C481)	[47]
Venetoclax	BCL2	*BCL2* mutations	[90,91]
		Mutations in *BTG1* and *BRAF*	[89]
		Deletion of CDNK2A/B	[89]
		Amplification of PD-L1	[89]
		MCL1 overexpression	[108]
		Mitochondrial reprogramming	[93]
Idelalisib	PI3-kinase-d	Increased IGF1R expression and MAPK-ERK1/2 activity	[68]
		Mutations in MAPK-ERK1/2 pathway (*KRAS, BRAF, MAP2K1*)	[69]

BTK: Bruton’s tyrosine kinase; FCR: fludarabine, cyclophosphamide, rituximab; BR: bendamustine, rituximab; ATM: ataxia telengiectasia mutated; MAPK: mitogen-sctivated protein kinase

Next-generation anti-CD20 monoclonal antibodies have also been developed to optimise the binding and cytotoxic effects towards B cells. Ofatumumab is a fully humanized, type I, CD20-specific monoclonal antibody. Ofatumumab binds to CD20 with greater affinity than does rituximab and has a slower dissociation rate^[29]^. Although, ofatumumab does not induce direct cell toxicity, the greater avidity of the antibody for CD20 means it is less affected by complement regulatory proteins and has greater CDC-mediated effects compared to rituximab^[30]^.

Obinutuzumab is a type II, anti-CD20 monoclonal antibody that has a distinct variable region and recognises a different CD20 epitope compared to rituximab. The high affinity of obinutuzumab for CD20 means it is a more potent inducer of direct cell death and ADCC than is rituximab. However, obinutuzumab is a less potent inducer of CDC, since the obinutuzumab-CD20 complex is unable to move into lipid rafts. Obinutuzumab has been shown to have activity in relapsed/refractory CLL patients^[31]^. In the German CLL study group (GCLLSG) CLL11 trial, patients with comorbidities were treated with chlorambucil alone, chlorambucil plus obinutuzumab or chlorambucil plus rituximab. This trial demonstrated that both the response and minimal residual disease (MRD)-negativity rate of patients treated with chlorambucil plus obinutuzumab were significantly higher than those of patients in the other arms. In terms of resistance related to *NOTCH1* mutations, as stated, ofatumumab appears to be similar to rituximab; however, there is some evidence to suggest that this resistance is overcome by obinutuzumab^[32,33]^. In the current era, treatment refractoriness or resistance to CIT modalities in CLL is most commonly addressed by a change in therapeutic approach to targeted therapies.

## Inhibitors of BCR-mediated signalling

**Figure 1:**
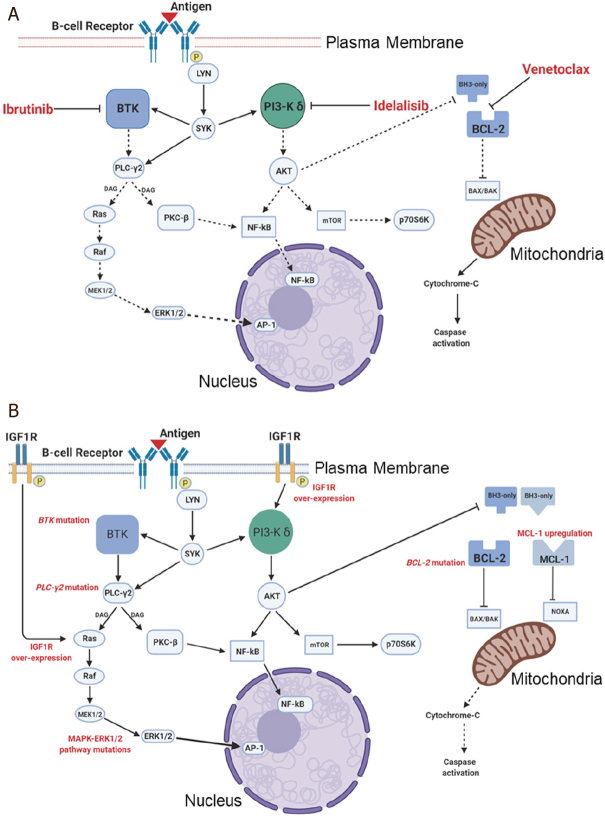
Schematic diagram illustrating the signalling pathways targeted by ibrutinib, idelalisib and venetoclax in CLL (A) and the known mechanisms of resistance to each of these agents (B) in CLL cells. CLL: chronic lymphocytic leukaemia; Btk: bruton's tyrosine kinase; LYN: Lck/Yes novel tyrosine kinase; AKT: protein kinase B; SYK: spleen tyrosine kinase; PKC: protein kinase C; BCL: B-cell Lymphoma; MCL-1: myeloid leukemia cell differentiation protein; mTOR: mammalian target of rapamycin

Tonic activation of the BCR signalling pathway is a hallmark of CLL cells and plays a key role in their survival and proliferation. Signalling via the BCR is activated through binding of specific antigens to the receptor and as a result of the interaction of CLL cells with a variety of other cell types, including stromal cells and T cells^[34]^. The BCR pathway also plays a crucial role in mediating the effects of a variety of chemokines and growth factors. Signalling via the BCR is mediated by a range of receptor-associated and intracellular kinases, which ultimately induce a protein expression profile consistent with B cell survival, proliferation, migration and adhesion^[35]^. A better understanding of the important roles of the BCR pathway and the tumour microenvironment in CLL has driven the clinical development of drugs that inhibit key signalling components within the BCR pathway. Table 1 and the schematic diagram, Figure 1, detail and illustrate the mechanisms of action and drug resistance associated with the therapeutic agents used in CLL to target the BCR signalling pathway and inhibit B-cell lymphoma 2 (BCL2).

A phenomenon common to the drugs described here is the rapid lymphocytosis observed in most patients following commencement of therapy. This effect is interesting from a biological perspective since it highlights the mechanisms by which CLL cells migrate to and are retained within the tumour microenvironment; BCR-targeted drugs alter the expression of chemokine receptors including CXCR4^[36,37]^ and adhesion molecules including CD49d^[38]^. The redistribution of leukemic cells from the lymph nodes is observed clinically as a rapid decrease in lymph node size with a concomitant rise in the circulating white cell count. In most patients this lymphocytosis is transient, since the circulating CLL cells no longer receive the support and proliferative drive conferred by the tumour microenvironment. The CLL tumour microenvironment may also play an important role in mediating resistance or reduced sensitivity to the BCR-targeted therapies discussed in the following section. The interaction of CLL cells with other cells present in the tumour microenvironment and certain cytokines and growth factors stimulate intracellular signalling pathways that promote CLL cell survival and counter the efficacy of targeted therapies. This is illustrated in a recent study by Kapoor *et al*.^[39]^, in which reduced sensitivity of CLL and diffuse large B-cell lymphoma (DLBCL) cells to ibrutinib was associated with increased activity of the PI3-kinase pathway.

## Inhibitors of Bruton’s tyrosine kinase

### Ibrutinib

Ibrutinib is an irreversible inhibitor of Bruton’s tyrosine kinase (BTK). BTK is a non-receptor tyrosine kinase, which is constitutively active in CLL cells as a result of tonic BCR-mediated signalling and activity of the kinases Lck/Yes novel tyrosine kinase (LYN) and spleen tyrosine kinase (SYK). Ibrutinib binds at the cysteine 481 (C481) residue within the kinase domain of BTK and prevents the phosphorylation of its downstream targets^[40]^.

As a single agent, ibrutinib has proven highly effective, initially in the treatment of relapsed/refractory CLL patients and more recently as a frontline therapy^[41,42]^. Adverse events, including atrial fibrillation, excessive bleeding, and arthralgia are common among CLL patients treated with ibrutinib^[43,44]^.

Despite high initial response rates to ibrutinib, a significant proportion of patients relapse with progressive disease. Treatment options for patients with disease transformation are very limited. Resistance to ibrutinib is more common among heavily pre-treated patients and is associated with mutations of the C481 residue in BTK, which permits only reversible binding of ibrutinib to the kinase^[45,46]^. Mutations of phospholipase-g2 (*PLC-g2*) that allow the circumvention of BTK and activation of signalling of the same pathway have also been associated with resistance to ibrutinib^[46]^. Similar to mutations of the C481 residue, the *V537I*, *T474A* and *L528W BTK* mutations identified in ibrutinib-resistant patients are all located within the kinase domain and are believed to interfere with the ability of ibrutinib to bind to the protein^[47]^. The *T316A* mutation documented in a single ibrutinib-resistant patient is distinct since it is located within the Src homology 2 (SH2) domain of BTK and confers resistance to ibrutinib by modifying intracellular signalling pathways rather than by interfering with ibrutinib binding^[48]^. The study proposed that mutations within the SH2 domain may interfere with the ability of BTK to interact with key intermediary proteins and to phosphorylate *PLC-g2*. In all these studies the *BTK* mutations were not evident prior to the patients developing ibrutinib-resistant disease, although another study demonstrated that *BTK* mutations frequently predate clinical resistance^[49]^. Collectively, the studies highlight the association between mutations in *BTK* or *PLC-g2* and ibrutinib resistance and reinforce the importance of this signalling pathway in the pathogenesis of CLL.

A relatively small proportion of patients develop ibrutinib resistance in the absence of detectable mutations of either *BTK* or *PLC-g2*. Although the exact mechanisms of resistance in these cases remain unclear, it can be speculated that mutations in signalling components downstream of BTK, in the mitogen-sctivated protein kinase (MAPK) pathway, for example, or dysregulation of another pro-survival pathway may also confer resistance to ibrutinib in CLL. This notion is supported by a study in which overexpression of programmed cell death protein-1 (PD-1) and BCL2 and increased activity of the AKT/mTOR pathway were observed in CLL patients who developed progressive disease or Richter’s transformation following treatment with ibrutinib^[50]^. Jain *et al.*^[51]^, demonstrated a similar mechanism in ibrutinib-resistant DLBCL cell lines and showed that survival of these lines was dependent on signalling via the phosphoinositol 3 kinase (PI 3-kinase) pathway. Since proteins of the BCL2 family are regulated by the activity of the PI3-kinase/AKT pathway, it is conceivable that increased PI3-kinase activity in CLL cells may account for the pro-survival shift in expression of the BAX and BCL2 proteins, which correlates with poor outcome following ibrutinib treatment^[52]^. Since the mechanisms of action of ibrutinib include downregulation of integrin expression, which liberates CLL cells from the tumour microenvironment, the expression of proteins involved in the retention of leukaemic cells within the lymph nodes may play a significant role in the response of patients to this drug. This was illustrated in the study by Tissino *et al*.^[53]^, in which expression of CD49d and activation of the integrin VLA-4 were associated with reduced nodal clearance and shortened progression-free survival in patients treated with ibrutinib. Irrespective of the mechanism, the fact that a significant proportion of patients develop ibrutinib-resistant disease underscores the importance of on-going trials of ibrutinib in combination with other agents, including venetoclax^[54]^ and umbralisib^[55]^.

### Next-generation BTK inhibitors

Trials of the next-generation BTK inhibitors acalabrutinib^[56]^, zanubrutinib^[57]^, tirabrutinib^[58]^ (NCT02983617, NCT02457598) and LOXO-305 (NCT03740529) are currently underway as single agents or in combinations for haematological diseases. In addition, efficacy of the BTK inhibitors evobrutinib^[59]^, ABBV-105^[60]^ and poseltinib^[61]^ has been assessed for multiple sclerosis, systemic lupus erythematosus and auto-immune diseases, respectively. Agents that selectively inhibit both BTK and other kinases have also been developed. Vecabrutinib is a reversible inhibitor of BTK that also targets interleukin-2-inducible tyrosine kinase while CG-806 is an inhibitor of both BTK and the FMS-like tyrosine kinase-3 (FLT-3). ARQ-531 inhibits BTK as well as a range of other kinases including LYN and MEK1. Many of these next-generation BTK inhibitors are reported to share the same mechanism of action as ibrutinib but were designed to be more specific for BTK with less effect against other members of the TEC family of kinases compared to ibrutinib. A study describing the biochemical effects of ibrutinib compared to those of tirabrutinib, acalabrutinib and spebrutinib has recently been published^[62]^. Many of these drugs, including the reversible BTK inhibitors LOXO-305, vecabrutinib and ARQ-531, also inhibit both wild-type and BTK harbouring mutations of the C481 residue. No data are available yet concerning whether these drugs are associated with a lower risk of bleeding or rates of atrial fibrillation, which are the main complications associated with ibrutinib^[63]^.

Currently, the limited follow-up data from trials of CLL patients treated with next-generation BTK inhibitors also means it is too early to determine the incidence of drug resistance among these patients. Acalabrutinib is a more potent and selective BTK inhibitor than ibrutinib and is believed to be associated with a lower rate of adverse events. The first published study reporting resistance among CLL patients treated with acalabrutinib identified 2 patients, one of whom developed resistant disease with a mutation in *BTK*, the other who developed a Richter’s transformation with a mutation in *BTK* and a dominant *TP53*-mutated clone^[47]^. The patients who developed progressive disease and a Richter’s transformation while on treatment with acalabrutinib both had mutations of the C481 residue in BTK. Given that the allele frequency of *BTK* and *PLC-g2* mutations is often low in BTK inhibitor-resistant patients, it seems likely that these mutations are only part of the story and that other genetic lesions or pro-survival mechanisms play a significant role in the resistance of patients to these drugs^[64]^.

## PI3-kinase inhibitors

### Idelalisib

Idelalisib is a specific inhibitor of the d-isoform of phosphoinositide 3’-kinase (PI3-kinase). All the PI3-kinase isoforms, a, b, g and d, mediate signals downstream of the BCR and play important roles in the survival and proliferation of CLL cells. However, the PI3K-d isoform is the predominant isoform expressed in hematopoietic cells and targeting this isoform has proven highly effective in CLL particularly for patients with relapsed/refractory disease^[65]^.

Despite promising initial results^[65]^, further clinical development of idelalisib has been limited by its high adverse event rate, most notably in the form of auto-immune-mediated colitis^[66]^. There are also a limited number of studies that have explored mechanisms of idelalisib-resistance in CLL (reviewed in study^[67]^). Although the exact mechanisms are unclear, a study of a CLL mouse model and primary CLL cells *in vitro* suggested that, while resistance to idelalisib is not associated with specific gene mutations, it may be linked to increased expression of insulin-like growth factor 1 receptor (IGF1R) and subsequent activation of the MAPK pathway^[68]^. The importance of increased IGF1R expression in this context was confirmed in the same study by demonstrating that idelalisib-resistant CLL cells were sensitive to a specific IGF1R inhibitor. A study by Murali *et al*.^[69]^ also demonstrated the association between increased MAPK-ERK1/2 signalling and resistance of CLL cells to PI3-kinase inhibition. However, in this study of primary CLL cells from idelalisib-resistant patients, the authors showed that drug resistance and constitutive MAPK pathway activation was associated with the acquisition of recurrent mutations in the *KRAS*, *BRAF* and *MAP2K1* genes^[69]^. Evidence of crosstalk between the MAPK and PI3-kinase signalling pathways was also evident in a study from our laboratory, which demonstrated that inhibition of MAPK-ERK1/2 signalling resulted in increased phosphorylation of AKT in primary CLL cells^[70]^. Under these conditions, it appeared that any cytotoxic effects of the MEK1/2 inhibitor binimetinib were countered by an increase in the phosphorylation of AKT. The ramifications of this are discussed in more detail in the later section on future therapeutic strategies for CLL.

### Duvelisib

Duvelisib is a specific inhibitor of both the g and the d isoforms of PI3-kinase. In the DUO phase III clinical trial, 313 patients with relapsed/refractory CLL were treated with either single agent duvelisib or ofatumumab^[71]^. The study demonstrated that duvelisib was effective as a treatment for relapsed/refractory CLL, even in patients with deletion of 17p, and is significantly more effective than ofatumumab^[71]^. However, due to the limited availability of clinical trial and follow-up data, there have been no published reports concerning duvelisib resistance among CLL patients.

It is conceivable that similar mechanisms that confer resistance to idelalisib may also be pertinent to duvelisib; constitutive activation of MAPK-ERK1/2 signalling, whether through increased phosphorylation of components of this pathway or activating mutations, may compensate for inhibition of PI3-kinase-mediated signalling and promote the survival of CLL cells. In a study of B- and T-cell lymphoma, resistance of cell lines to duvelisib and the pan-PI3-kinase inhibitor copanlisib was associated with increased expression of interleukin 6, which resulted in increased phosphorylation of several intracellular signalling molecules, including STAT, AKT, p70S6K, MAPK and NF-κB^[72]^. It remains likely that in CLL, resistance to specific inhibitors of PI3-kinase-mediated signalling may also be mediated by as yet undescribed mechanisms that involve compensatory upregulation of alternate pro-survival pathways.

### Umbralisib

Single-agent trials of the dual PI3-kinase-d and casein kinase-1e (CK-1e) inhibitor umbralisib, either alone or in combination with other drugs are also being conducted for CLL. In a phase I study of umbralisib in 90 patients with CLL, B-NHL or Hodgkin’s lymphoma, the incidence of auto-immune adverse events, particularly colitis, was significantly less than previously observed in trials of idelalisib^[73]^. Among the 20 CLL patients on this trial, for which response data were available, 85% (17) had an objective response to treatment. To date, there have been no reports concerning the mechanisms of resistance to umbralisib, but considering its favourable toxicity profile compared to idelalisib and with on-going trials of umbralisib in combination with other agents, studies detailing umbralisib-refractory disease may emerge.

## Other BCR-signalling inhibitors

Inhibitors targeting other components of the BCR signalling pathway have also been or are currently being trialled for CLL. The SYK inhibitor fostamatinib and the dual SYK/Janus kinase 1/3 (JAK1/3) inhibitor cerdulatinib have both been studied. Among 11 patients with relapsed/refractory CLL treated on a phase I/II study of fostamatinib the overall response rate was 55% but the rate of dose-limiting toxicities was high and included diarrhoea, neutropenia and thrombocytopenia^[74]^. In a phase I trial of cerdulatinib that enrolled 8 patients with relapsed/refractory CLL, 2 patients achieved a clinical response^[75]^. Two patients who had previously progressed on therapy with ibrutinib and then progressed while on cerdulatinib were found to harbour the C481 mutation in *BTK* as well as mutations of *TP53* and *EP300*, which encodes a histone acetyltransferase. A recent *in vitro* study demonstrated that cerdulatinib may be effective in overcoming ibrutinib resistance and the supportive effects of the tumour microenvironment^[76]^, suggesting that resistance to cerdulatinib in the trial by Hamlin *et al*.^[77]^ may be related to the mutation of *TP53* or dysregulation of transcription due to mutation of *EP300*. Further data are likely to become available when the results of an on-going phase I/IIa trial of cerdulatinib are presented (NCT01994382). Since these trials are comprised of relapsed/refractory and generally heavily pre-treated patients, the response rates are encouraging and suggest that inhibitors targeting alternate components of the BCR-signalling pathway may be effective for some patients with extremely poor risk disease. While it is difficult to draw any conclusions regarding the mechanisms of drug resistance in patients who experienced disease progression in these trials, it is conceivable that the same mechanisms already discussed as being associated with resistance to the BTK and PI3-kinase inhibitors would be relevant to these drugs.

Protein kinase C (PKC) also plays a significant role in BCR signalling and the survival of CLL cell^[78]^. Preclinical studies of enzastaurin suggest that specific inhibition of the PKC-bb isoform may have efficacy in CLL^[79]^. While there are no reports of clinical trials of enzastaurin in CLL, an on-going phase I trial of MS-553 (NCT03492125), will determine whether the drug is tolerable and whether inhibition of PKC-b may represent another therapeutic strategy for refractory CLL.

## Inhibition of BCL-2-family proteins

CLL cells constitutively overexpress the BCL2 protein. The BCL2 family of proteins comprises proteins that have roles in promoting either cell survival or apoptosis^[80]^. The members of this family with pro-survival functions include BCL2, BCLw, BCLxL, MCL1 and BFL1, while a subset of this group, termed BH3-only proteins, which include BAD, NOXA, BIM, PUMA and tBID, bind to and inhibit pro-survival members of the family. Along with these pro-survival and inhibitory proteins BAX and BAK function as pro-apoptotic effectors by inducing the depolarisation of the outer mitochondrial membrane, resulting in cytochrome C release and a caspase-dependent apoptotic signalling cascade. Under normal cellular conditions, the balance of proteins in the BCL2 family is tightly regulated, but in several disease conditions, including in CLL, unchecked cell survival and proliferation results from a dramatic shift in this balance. Overexpression of BCL2 in CLL has been linked to low expression of miRs including miR-15a and miR-16-1, which are known to repress BCL2 expression^[81]^. As CLL-cell survival is dependent on overexpression of BCL2, the protein is an attractive target for therapy.

The first drug in a class known as the BH3-mimetics, ABT-263 or navitoclax, was found to be effective *in vitro* against a range of cell types, including CLL, and *in vivo* against solid tumours, including small-cell lung carcinoma^[82,83]^. Interestingly, the study by van Delft *et al*.^[82]^, identified several cell lines and a mouse model of lymphoma that were resistant to navitoclax due to the fact the drug does not inhibit MCL1. In clinical trials, navitoclax had significant antitumour activity but also caused profound thrombocytopaenia due to inhibition of BCLxL^[83]^. The promising antitumour effects of navitoclax prompted the development of other drugs in this class, most notably venetoclax.

### Venetoclax

Venetoclax (ABT-199) was developed to specifically inhibit BCL2 with only weak activity towards other members of the BCL2 family, including BCLxL thereby minimising thrombocytopaenia as a dose-limiting toxicity^[84]^. In addition to frequent clearing of normal and CLL B cells from the peripheral blood and lymph nodes, venetoclax therapy also significantly reduces the number of regulatory T cells (Tregs) and TNF-α- and IFN-λ-producing CD8^+^ T-cells and significantly increases IFN-λ production from NK cells^[85]^. These observations suggest that venetoclax has both direct cytotoxic and immunomodulatory effects. Interestingly, although the venetoclax mechanism of action is TP53-independent^[86]^, patients harbouring TP53 dysfunction have a poorer outcome in clinical trials^[87,88]^.

Despite its efficacy, most heavily pre-treated CLL patients will relapse during or following treatment with venetoclax. Whole-exome sequencing of samples from 8 patients, taken before initiating venetoclax treatment and after the patients developed resistance to the treatment, identified lesions in several cancer-related genes^[89]^. Mutations of *BTG1* and *BRAF*, deletions of *CDNK2A/B* and amplification of PD-L1 were all identified in samples from venetoclax-resistant patients, suggesting there may be multiple mechanisms that confer resistance to venetoclax in CLL. A study by Blombery *et al*.^[90]^ identified a G101V mutation of *BCL2* in samples from 7 of 15 venetoclax-resistant patients. However, the mechanisms that conferred resistance to the drug in the remaining 8 patients in this study were not elucidated. In another recent study, Tausch *et al*.^[91]^ identified one venetoclax-resistant patient with a D103Y mutation in BCL2, prior to the acquisition of a G101V mutation. In this study 3 of the 4 venetoclax-resistant patients were found to have the G101V mutation. Both studies suggest that the G101V and D103Y mutations interfere with the binding of venetoclax to BCL2. As these mutations were only present after > 2 years of therapy, it is likely that they were acquired during treatment rather than harboured by the leukemia cells prior to treatment, which is supported by the fact that they were undetectable in all patients prior to commencing treatment. Interestingly, neither mutation of *BCL2* was detected in any of the samples from the 8 patients studied by Herling *et al*.^[89]^, providing further evidence that these mutations do not account for every case of venetoclax-resistant CLL.

The notion that alternate pro-survival mechanisms may also play a significant role in conferring resistance to venetoclax is illustrated by preclinical studies of acquired resistance in lymphoma cell lines^[92,93]^. These studies demonstrated that resistance was associated with increased expression of BCLxL and MCL1, an increase in the activity of the PI3-kinase pathway and changes in cellular energy metabolism. These studies also highlight how understanding the mechanisms of resistance can be harnessed to rationalise novel drug combinations; Choudhary *et al*.^[92]^ demonstrated that venetoclax-resistant NHL cell lines could be sensitised to the BH3-mimetic by combining the drug with inhibitors of the PI3-kinase signalling pathway. Increased expression of alternate BCL2 family proteins in CLL cells resulting from the interaction of the leukaemic cells with other cell types that comprise the tumour microenvironment is also likely to play an important role in determining the response of patients to venetoclax. This was demonstrated in the study by Vogler *et al*.^[94]^, which showed that CLL cells co-cultured with stromal cells were 1000-fold less sensitive to ABT-737, a predecessor to venetoclax. It is conceivable that the sensitivity of CLL cells to venetoclax may also be related to changes in the expression of BCL2 and other proteins of the BCL2 family. A preclinical study of venetoclax in multiple myeloma demonstrated that the efficacy of venetoclax is dependent on high expression of BCL2 but also low expression of BCLxL and MCL1^[95]^. Similar findings were observed in a phase I trial of venetoclax for multiple myeloma; venetoclax was particularly effective for patients with a translocation between chromosomes 11 and 14 (t11:14), which occurs in approximately 20% of patients and is associated with higher levels of BCL2 expression in relation to other BCL2 family proteins^[96]^.

## Overcoming or avoiding drug resistance in CLL

### Combination strategies

A better understanding of the mechanisms of drug resistance in CLL has highlighted several situations in which targeting two or more signalling pathways may be extremely effective and may avoid the development of drug resistance or disease transformation. In addition, synergy between different drugs may have the potential to reduce the toxicity associated with a single drug without compromising efficacy. This concept has been the focus of numerous preclinical and clinical studies.

The addition of anti-CD20 antibodies to established therapies has historically been associated with improved clinical efficacy. An initial study combining ibrutinib with rituximab showed promising results in terms of safety and activity for CLL patients with relapsed/refractory disease^[97]^; however, contradictory studies have since shown that antagonism occurs between ibrutinib and anti-CD20 antibodies. This antagonism stems from observations that ibrutinib therapy reduces CXCR4-mediated signalling, resulting in decreased expression of CD20 and impairs T cell function, which reduces the ADCC-mediated effects of rituximab^[37,98]^. Ibrutinib-induced downregulation of CD20 is reversible, and therefore, the efficacy of this combination treatment might depend on the sequence of administration. This hypothesis is supported by a study in which the response rate to sequential administration of ibrutinib and ofatumumab was 100% compared to 71% when the sequence was reversed or 79% when both drugs were administered simultaneously^[99]^. A recent study has shown that ibrutinib and rituximab may be effective in combination when the drugs are given simultaneously when rituximab is administered initially at a small dose but then ramped up over the initial 3 cycles^[100]^. Aside from these considerations regarding drug sequencing, follow-up studies are required to determine the long-term efficacy of ibrutinib in combination with rituximab. It has been surmised that the efficacy of this regimen may be limited by the fact that both drugs are most active against CLL cells with active BCR signalling and the highest levels of CD20 expression^[37]^.

Studies suggest that patients who develop ibrutinib-resistant disease respond well to venetoclax^[101]^, which raises the possibility that early identification of patients with mutations of *BTK* or *PLC-g2* and intervention with venetoclax may be an effective strategy. However, given the dismal prognosis and lack of effective salvage strategies for patients who develop a Richter’s transformation, it would seem logical that strategies that avoid drug resistance and disease transformation would be more effective than salvage therapies. This notion is supported by recent data from clinical trials, including the phase II trial CLARITY of ibrutinib and venetoclax for CLL patients with relapsed/refractory disease^[102]^, which demonstrated that undetectable MRD status was achieved in 53 and 36% of patients, in blood and bone marrow, respectively. In a similar study by Jain *et al*.^[103]^, 88% of patients achieved a complete remission (CR) or complete remission with incomplete haematological recovery (CRi), and 61% were in remission with undetectable MRD in their bone marrow, which is significantly higher than the 6.2%-25.8% CR rate observed among patients treated with ibrutinib alone^[104]^.

Despite the limitations related to its toxicity, the efficacy of trials of idelalisib demonstrated the therapeutic potential of targeting PI3-kinase in CLL^[105]^. These studies provided a strong rationale for further studies of PI3-kinase inhibitors, including the recently published phase I/Ib trial of the dual PI3-kinase-d and casein kinase-1e (CK-1e) inhibitor umbralisib in combination with the CD20 monoclonal antibody ublituximab in patients with CLL and B-NHL^[106]^. The overall response rate among 22 CLL patients with relapsed/refractory disease, including 9 (41%) with deletion of *TP53*, was 62%. Although these data are encouraging, at the time of analysis, 67 of the 75 (89%) patients on this trial had discontinued therapy, primarily due to disease progression (59%).

Trials of umbralisib in combination with ibrutinib have also been conducted in CLL and mantle cell lymphoma^[55]^. As already mentioned, the PI3-kinase-d and CK-e inhibitor, umbralisib may represent an alternative, less toxic means of targeting PI3-kinase-mediated signalling compared to idelalisib. In combination with ibrutinib, with or without ublituximab, umbralisib may block the activation of alternate pro-survival signals that arise in CLL cells treated with either drug alone^[55,106]^. Data from the phase I/Ib trial by Davids *et al*.^[55]^ suggest that this is a well-tolerated regimen with an overall response rate of 90% among 42 patients with relapsed/refractory CLL or mantle cell lymphoma. Extended follow-up of patients on this trial or additional studies are required to determine the incidence and mechanisms of drug resistance among patients treated with this regimen.

Trials of venetoclax in combination with CD20-targeted therapies are currently on-going. Although it is unclear whether addition of rituximab has any benefit over venetoclax monotherapy for relapsed/refractory CLL patients^[107]^, the rationale for these trials is supported by preclinical data. A study of venetoclax in combination with rituximab or obinutuzumab demonstrated that the CD20 antibodies countered resistance to venetoclax induced by co-culture of the CLL cells with a stromal layer^[108]^. Previous studies from the same group suggest that this increase in sensitivity is related to the ability of the anti-CD20 antibodies to induce apoptosis-independent cell killing of CLL cells, which has been stimulated by co-culture with stromal cells^[109]^.

### Future therapeutic strategies

Trials of ibrutinib and the next-generation BTK inhibitors demonstrate the potential of targeting this pathway in CLL. A recent study suggests that targeted degradation of BTK may also be a strategy for inactivating this kinase and is effective against BTK harbouring mutations at the C481 residue^[110]^. Dobrovolsky *et al*.^[110]^ demonstrated that their lead compound, DD-03-171, degrades not only BTK but also the transcription factors IKZF1 and IKZF3, effectively blocking BCR-mediated signalling and inhibiting the proliferation of leukaemic cells from patients with mantle cell lymphoma. The IKZF transcription factors are targets of the immunomodulatory (IMiD) class of drugs and putative driver mutations of *IKZF3* occur in approximately 2% of CLL patients^[8]^.

We and others have demonstrated that paradoxical activation of an alternate pro-survival pathway can occur in CLL cells treated with some inhibitors^[70,111,112]^. In these studies, inhibition of MEK1/2 in the MAPK-ERK1/2 signalling pathway resulted in increased phosphorylation of AKT. These observations raise the possibility that there may be multiple, as yet undefined situations under which crosstalk between pathways may compensate when one pro-survival pathway is blocked and that these mechanisms confer resistance to specific kinase inhibitors. Elucidating the network of crosstalk may help to better understand the mechanisms of drug resistance and identify rational combinations of drugs. This notion is illustrated by our recent studies demonstrating that inhibition of MEK1/2 by binimetinib is effective against CLL cells when combined with an AKT inhibitor^[111]^ or a BH3-mimetic^[70]^. The efficacy of similar rational drug combinations has been demonstrated in preclinical studies, including studies showing that inhibition of MCL1 sensitises DLBCL, follicular lymphoma, mantle cell lymphoma^[113]^ and acute myeloid leukaemia^[114]^ cells to venetoclax.

Another therapeutic avenue currently under investigation is inhibition of PD-1, which is involved in immune surveillance of multiple forms of cancer, including CLL. A trial of the PD-1 inhibitor pembrolizumab demonstrated that the drug has activity in patients with a Richter’s transformation of CLL^[115]^. The response of these patients correlated with pre-treatment levels of PD-1 and its ligand PD-L1 in the patients lymph nodes and on the CLL cells, respectively^[115]^. Given that a significant proportion of CLL patients develop a Richter’s transformation, the objective response rate of 44% in this trial is promising and suggests that PD-1 blockade with pembrolizumab or the PD-1 antibody durvalumab^[116]^ may represent a promising treatment strategy for patients with aggressive disease or disease transformation.

Finally, single drugs that target multiple key signalling pathways may represent an effective means of preventing the evolution of drug resistant CLL clones. Inhibition of the molecular chaperone HSP-90, for example, is known to have significant effects on several key signalling pathways^[117]^. A more targeted approach is illustrated by our studies of the dual PIM and PI3-kinase inhibitor, IBL-202^[118]^, and by studies of the dual SYK/JAK inhibitor cerdulitinib, which is currently in a clinical trial^[75]^; *in vitro* studies of both IBL-202 and cerdulitinib^[76]^ demonstrate that these drugs are effective against CLL cells under conditions known to confer resistance to drugs in current clinical use, including ibrutinib and venetoclax.

## Concluding remarks

During the last two decades, there have been significant advances in the therapeutic options for CLL. Although CIT has proven effective, particularly for a subset of patients with markers of good prognosis, the increasing age demographic in many countries means that there is an increasing need for treatment strategies for the elderly, comorbid CLL patients. Targeted therapies are potentially less toxic, may lower secondary malignancy rates and may represent strategies for disease control rather than cure, compared to genotoxic regimens for elderly patients. However, disease relapse and drug resistance are still common among patients treated with BCR-signalling and BCL2 inhibitors, since the development of each novel signalling inhibitor has heralded new mechanisms of drug resistance. A better understanding of these resistance mechanisms should provide the rationale for new strategies to treat refractory disease or to minimise the risk of drug resistance or disease transformation.

## Declarations

### Availability of data and materials

All data sources are referenced in the manuscript and were sourced from online journal repositories.

### Copyright

© The Author(s) 2020.
